# Histological Analysis of Acetabular Bone Impaction Grafting According to Slooff 15 Years After Implantation

**DOI:** 10.1155/cro/2671931

**Published:** 2026-09-17

**Authors:** L. P. Weimer, G. Beckers, S. Milz, B. M. Holzapfel, D. Simon

**Affiliations:** ^1^ Department of Orthopaedics and Trauma Surgery, Musculoskeletal University Center Munich (MUM), University Hospital, LMU Munich, Munich, Germany, uni-muenchen.de; ^2^ Department of Anatomy, Ludwig-Maximilian University (LMU), Munich, Germany, uni-muenchen.de

**Keywords:** case report, protrusion, Slooff, total hip revision arthroplasty

## Abstract

**Case:**

We describe the case of a 78‐year‐old multimorbid male patient with a history of right‐sided total hip arthroplasty in 1996 and homologous acetabular bone graft reconstruction according to Slooff in 2009. In December 2024, the patient presented with acute right hip pain due to acute on chronic loosening of the implant. Diagnostic workup revealed a low‐grade periprosthetic joint infection with *Cutibacterium acnes*. After preoperative optimization, the patient underwent implant explantation and conversion to a Girdlestone state. Histological analysis of the explanted Slooff augment showed nonvital and nonincorporated cancellous bone, lacking osteocytes within the bone lacunae, indicating the absence of viable bone and remodeling.

**Conclusion:**

Reconstruction of acetabular defects, particularly in cases of severe bone loss or medial wall protrusion, remains a significant challenge in revision total hip arthroplasty (rTHA). The Slooff technique, involving morselized homologous bone grafts impacted into the acetabular defect and stabilized with a cemented cup, has been widely adopted for such cases due to its relative simplicity and promising midterm outcomes. However, the biological and mechanical long‐term integration of such constructs, as observed in this case, remains a topic of ongoing debate.

## 1. Introduction

Acetabular loosening following total hip arthroplasty (THA) is a challenging clinical problem, particularly in elderly patients with significant comorbidities [1]. The Slooff technique for acetabular reconstruction using homologous bone grafts has shown promising long‐term outcomes in selected cases with acetabular defects and protrusion [2–4]. However, revision surgery in multimorbid patients carries substantial risk, especially in the context of chronic infections [5].

Although the Slooff technique remains a viable option for managing acetabular defects and protrusions, particularly in the context of revision THA, concerns about the long‐term biological activity and osseous integration of cemented bone grafts are warranted [6]. In the revision setting, the osteogenic capacity of the surrounding host bone is usually impaired, resulting in an overestimation of the biological potential of such constructs. Careful patient selection, realistic outcome expectations, and exploration of biologically active alternatives are essential for improving long‐term success.

We therefore present the case of a patient with failure of the Slooff graft due to periprosthetic joint infection. To the best of our knowledge, this is the first description of an explanted and analyzed Slooff graft after 15 years in situ. The patient was informed about the analysis of the explant and provided his consent. This case report adheres to the CARE guidelines, and the completed CARE checklist has been provided as supporting information.

## 2. Case Report

**Figure 1:**
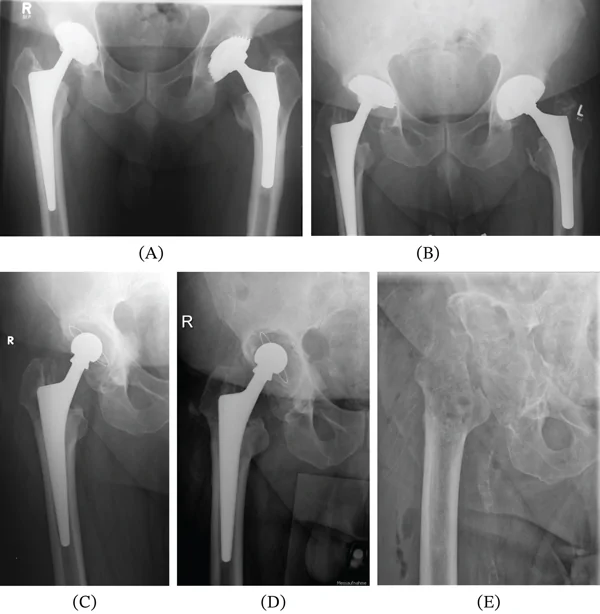
(A) Anteroposterior pelvic radiographs obtained in 2001. (B) Anteroposterior pelvic radiographs obtained in 2009 with cup loosening on the right side and performed cup revision on the left. (C) Anteroposterior pelvic radiographs obtained in 2011. (D) Anteroposterior pelvic radiographs obtained in 2014 with gradual loosening of the Slooff and increasing cup inclination. (E) Anteroposterior pelvic radiographs obtained in 2024 after explantation and conversion to a Girdlestone situation.

A 78‐year‐old male patient presented to our university emergency department in December 2024 as a referral from a rural hospital with acute, immobilizing right hip pain. His complex medical history included chronic obstructive pulmonary disease (COPD), obstructive sleep apnea syndrome (OSAS) treated with nCPAP, polycystic kidney disease with chronic kidney failure requiring hemodialysis, heart failure with preserved ejection fraction (HFpEF), hypertension, obesity, hemophilia A, chronic venous insufficiency with lower leg ulcers, and a history of colon carcinoma and gastrointestinal (GI) bleeding. The patient had previously undergone primary THA on the right side in 1996, and received homologous bone graft acetabular reconstruction using the Slooff technique in August 2009 at our center due to aseptic cup loosening (Figure 1A–C). Initial presentation at the referring hospital had raised suspicion of implant failure, which was confirmed via x‐ray (Figure 1D) and CT scan at our center. Laboratory and hematologic evaluations were performed, taking into account the patient′s hemophilia A, and a diagnostic joint aspiration revealed a leukocyte count of 9333/*μ*L. Microbiological analysis detected *Cutibacterium acnes*, indicating chronic periprosthetic joint infection. Following multidisciplinary discussion and preoperative optimization, including dialysis and hematologic management, explantation of the THA and conversion to a Girdlestone situation were performed on December 11, 2024 (Figure 1E)

Ten days after surgery, the patient developed severe influenza with a secondary bacterial infection, necessitating intubation and intensive care. The clinical course was complicated by hemorrhagic shock due to lower GI bleeding in the context of ischemic colitis and hemophilia A. During the course of his intensive care treatment, the patient received a total of 50 units of packed red blood cells, 8 FFP, 4 PPSB, and 4 g fibrinogen transfusions with daily substitution therapy with recombinant Factor VIII (Elocta).

Colonoscopy and gastroscopy on December 25, 2024, excluded upper GI bleeding, raising suspicion of ischemic colitis in the descending and sigmoid colon. Due to ongoing bleeding, an open left hemicolectomy with transverse colon resection and formation of an enterostoma was performed on January 7, 2025. Secondary peritonitis occurred due to suspected anastomotic leakage. Diagnostic ascites puncture on January 14, 2025, showed elevated neutrophils, with detection of *E. faecium* and *Candida albicans*. Broad anti‐infective therapy was administered, but additionally, the patient experienced duodenal ulcer bleeding with a Forrest Ib bleeding from a Dieulafoy ulcer, treated with clips and fibrin glue, and an in‐hospital cardiac arrest due to hypoxia on January 6, 2025, attributed to aspiration pneumonia. Therefore, despite maximal medical, surgical, and intensive care support, the patient′s condition continued to deteriorate. He died 4 weeks after surgery in early February 2025. The explanted Slooff graft and prosthesis were subjected to detailed histopathological analysis with prior informed consent of the patient. A structured patient timeline is attached in the supporting material.

**Figure 2:**
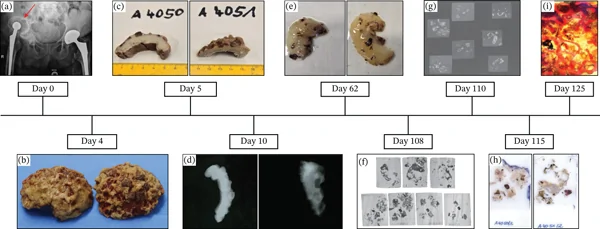
(a–i) Chronological assessment order of the Slooff graft for histomorphology. The numbers below the images indicate postexplantation days.

**Figure 3:**
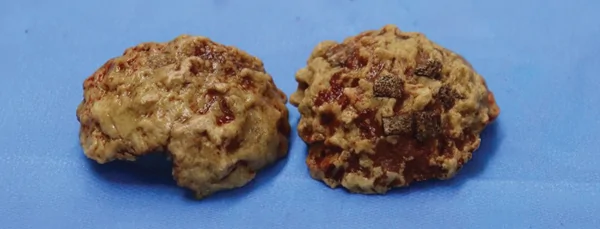
Explanted Slooff homologous bone graft and cement reconstructions of the acetabulum with clearly visible allogenic spongiosa chips in the PMMA‐cement.

The Slooff graft was explanted in December 2024 (Day 0, Figure 2a), thus 15 years after initial implantation. Macroscopically, the graft consisted of solid blocks of bone and cement (Figures 2b and 3) and was fixed in 50% ethanol at 4°C. For histological processing, two tissue cement blocks of approximately 4.5 × 0.8 cm were cut with a diamond bone saw (Cut‐Grinder, Walter Messner GmbH, Oststeinbek, Germany) and stored in 70% ethanol at 4°C (Figure 2c). Contact radiographs (Cabinet X‐ray System, Faxitron Series, Hewlett‐Packard Company, McMinnville, Oregon, United States) were obtained from both tissue samples with 40 kV tube voltage and an exposure time of 80 s (Figure 2d). The tissue samples were embedded in methyl methacrylate (MMA), and this involved a dehydration, defatting, and solvent exposure protocol. During the dehydration process, both sample blocks degraded into a spongy, slimy mass after first being placed in xylene on February 7, 2025 (Figure 2e). This usually indicates that a MMA consisting bone cement was used during the initial operation. Standard protocol was pursued further and embedding of the tissue rests was completed on March 13, 2025. Using a saw microtome (Saw Microtome Leica SP1600, Leica, Nussloch, Germany), both blocks were cut in serial sections with a thickness of approximately 200 *μ*m (Figure 2f). Again, contact radiographs of all sections (Figure 2g) were obtained, and then, the sections were glued to plastic slides before being ground and polished using an EXAKT 400CS grinding system (EXAKT, Norderstedt, Germany). Staining with Giemsa‐eosine was done prior (Figure 2h) to histological examination. The stained sections were microscopically assessed using a Zeiss Axiophot microscope (Zeiss, Göttingen, Germany). Pictures were obtained at fivefold magnification with a digital camera attached to the microscope (Zeiss AxioCam HRc), as exemplary shown in Figure 2i.

**Figure 4:**
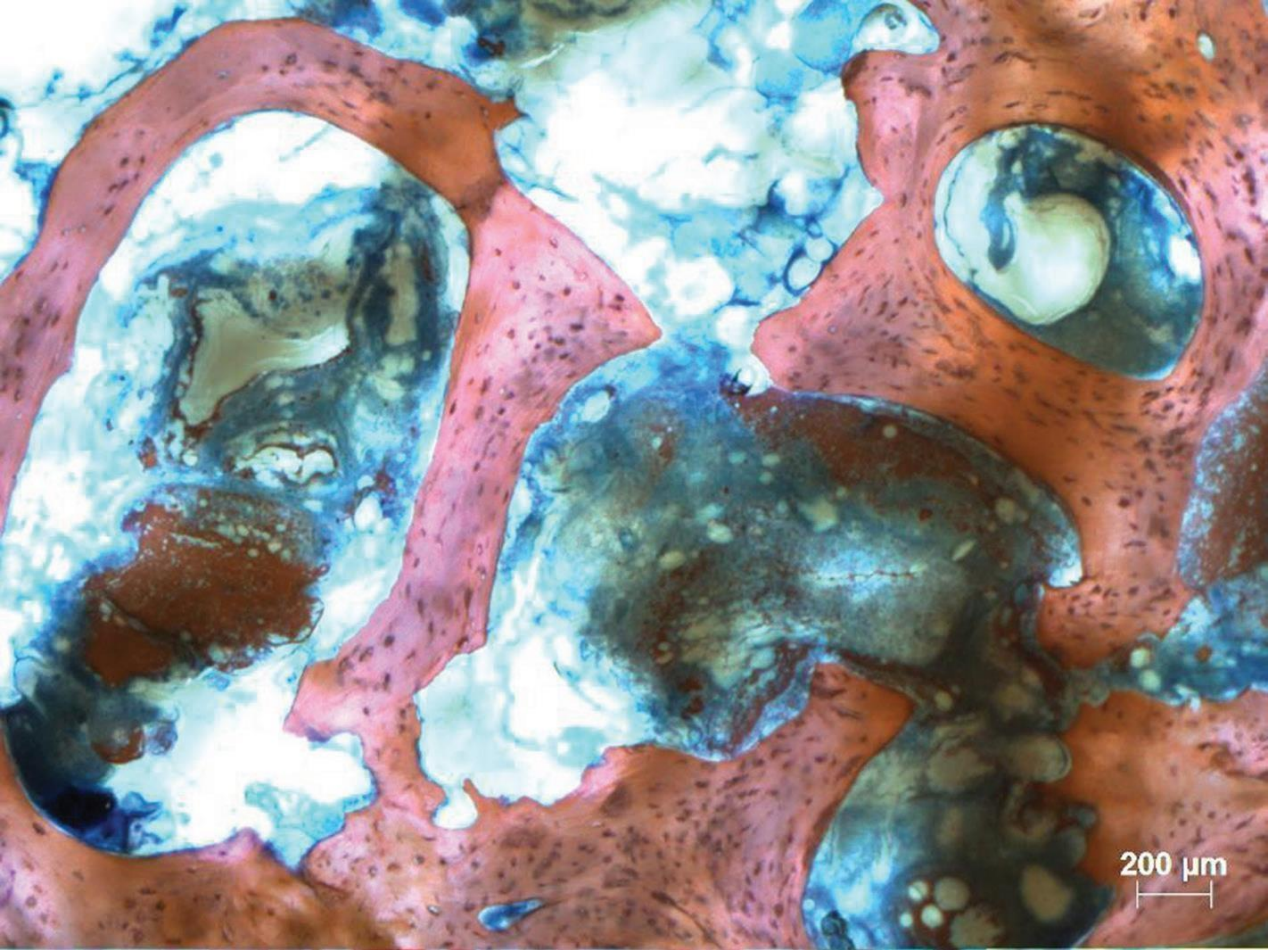
Histological image of the Slooff graft after Giemsa‐eosine staining showing fragmented, nonvital cancellous bone (pink) with intermittent amorphous bruising or hematoma (turquoise). Scale bar: 200 *μ*m.

**Figure 5:**
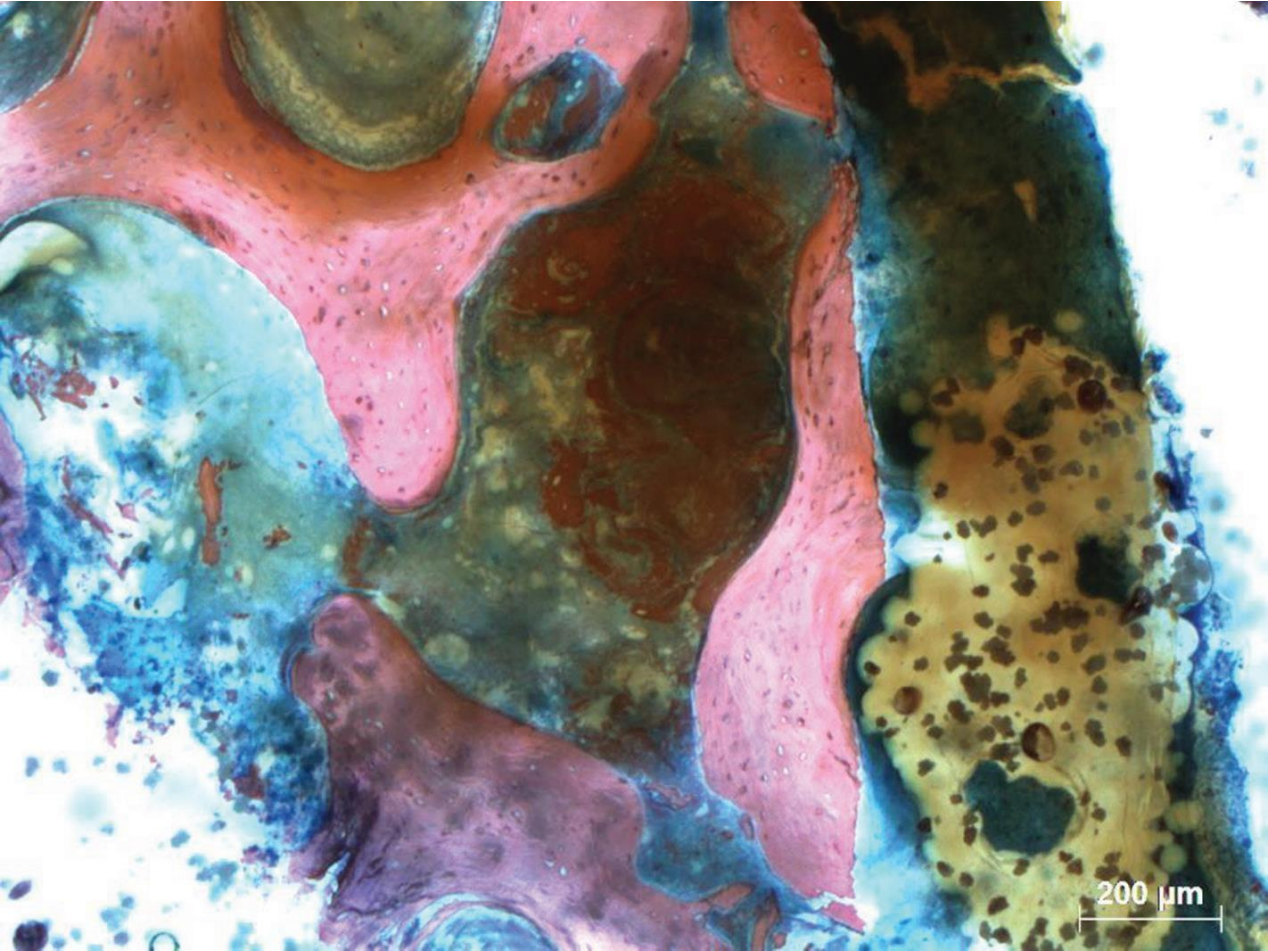
Histological image of the Slooff graft after Giemsa‐eosine staining. In addition to fragmented, nonvital cancellous bone (pink) with intermittent amorphous bruising or hematoma (turquoise) gray particles can be identified on the bottom right that are most likely related to zirconium dioxide from bone cement. Scale bar: 200 *μ*m.

**Figure 6:**
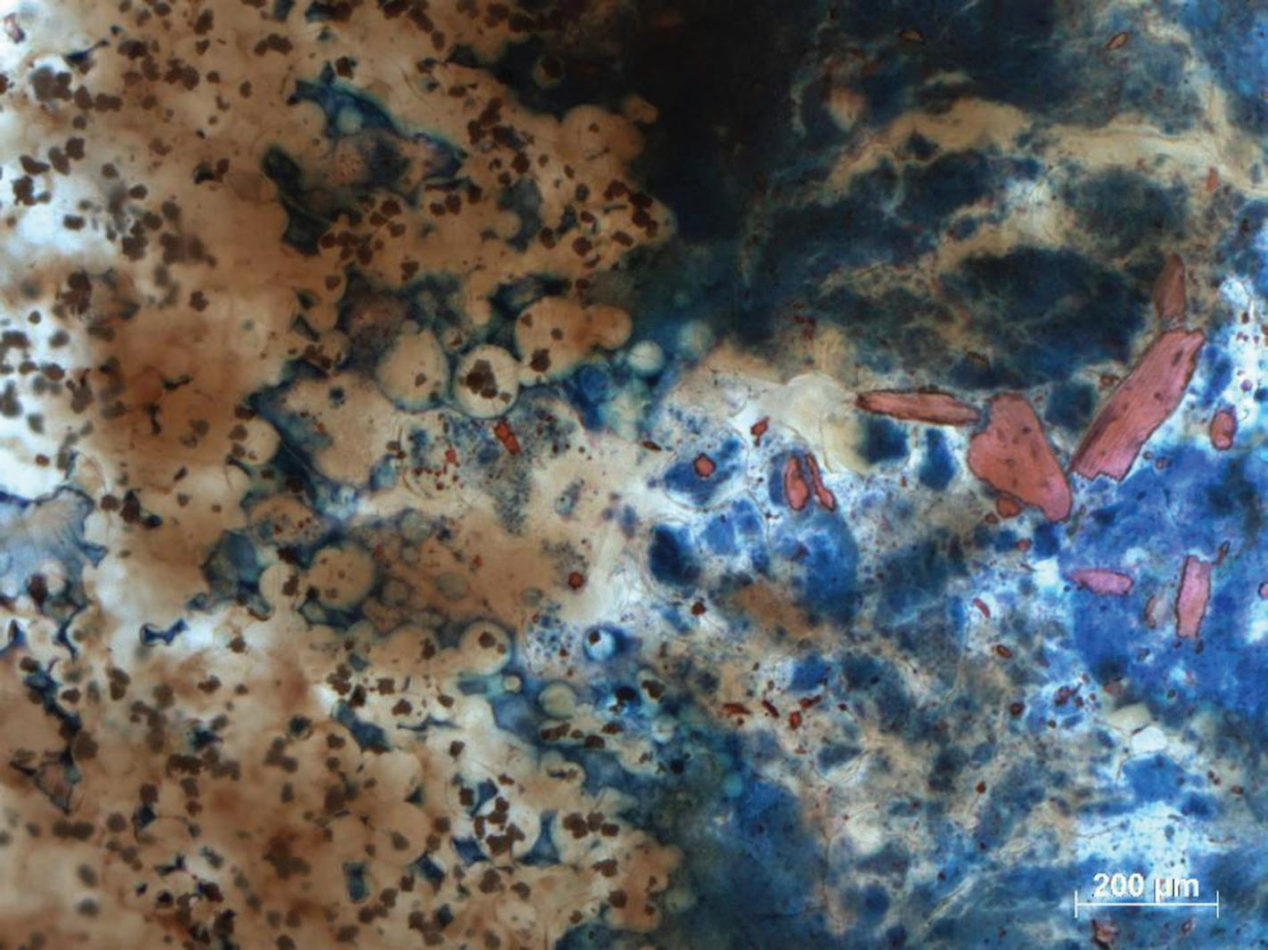
Histological image of the Slooff graft after Giemsa‐eosine staining. The matrix on the left is most likely associated with blood (brown–yellow), zirconium dioxide (dark‐gray particles) and intermittent air pocket inclusions (dark regions). Scale bar: 200 *μ*m.

**Figure 7:**
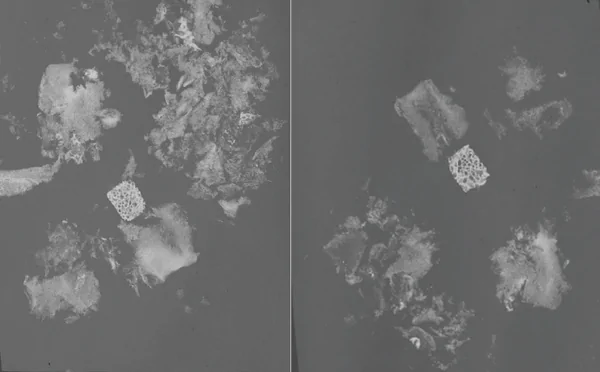
Exemplary plain radiographs of the Slooff graft section after cutting. Large radiopaque structure in the center of the images shows conglomerate of bone chips (note the fairly large cancellous fragment in the middle of the image) and bone cement. Intermittent formless radiopaque structures scattered among the image indicate bone cement insertions.

Histomorphological analysis showed predominantly fragmented, nonvital cancellous bone, which can be identified according to its pink staining in Figures 4 and 5. Among all sample sections, no osteocytes could be identified within the bone lacunae, indicating the absence of viable bone. Although remodeled spongiosa was detected, surrounding bone marrow edema with visible precipitation of mineral salts can be interpreted as sign of chronic irritation or infection. The brownish material shown in Figure 5 most likely resembles blood with intermittent dark air bubble inclusions. The amorphous blue material also shown in Figures 4 and 6 shows no indications of active biological tissue and most likely resembles fibrin or other protein residues (e.g., hematoma rests from the explantation operation). Gray particles that can be recognized ubiquitously in all sections, most likely are associated with zirconium dioxide, which is a radiopaque component of the used bone cement. This interpretation correlates with contact radiographs (Figure 7), as the corresponding surfaces are radiopaque.

## 3. Discussion

This case report demonstrates the clinical course and loosening of a homologous bone graft according to the Slooff technique. We furthermore analyzed the explant using histological techniques. Importantly, the present observation should be interpreted as a multifactorial failure scenario rather than as isolated evidence of graft‐ or technique‐related failure.

The Slooff method offers several advantages such as the immediate mechanical stability through impaction of bone grafts and cement fixation or the restoration of bone stock, which is said to be particularly beneficial in younger or more active patients, or in cases requiring future revision. In addition, the technical accessibility, with a reproducible surgical approach that does not necessarily require expensive and complex implants or cages, as well as the medial wall reconstruction, allowing for containment of the acetabular component even in cases of severe protrusion, are beneficial. In cases of acetabular protrusion, where the medial wall is compromised and intrapelvic migration of the cup may occur, the technique allows for structural containment using graft material while avoiding more complex alternatives such as custom triflange components.

Despite its advantages, the Slooff technique is not without significant limitations, especially with regard to biological integration and long‐term durability. In the present case, two groups of mechanisms should be separated. First, patient‐specific systemic factors may have substantially reduced the patient′s capacity for graft incorporation. These included hemophilia with relevant bleeding risk and hematoma formation, dialysis‐dependent chronic kidney disease with impaired mineral and bone metabolism, heart failure, obesity, COPD, chronic venous insufficiency, and the overall multimorbid condition. Such factors may independently compromise immune competence, vascularity, bone quality, and the ability to remodel allogenic bone. Second, graft‐ and technique‐related factors may have contributed locally, including the revision setting, acetabular bone loss, incomplete host‐graft contact, the interposition of polymethyl methacrylate (PMMA) cement, the heat generated during cement polymerization, and a possible mechanical shielding or sealing effect that may limit vascular invasion and cellular migration. Accordingly, the absence of osteocytes within the graft and the only partial remodeling observed in this case should be understood as the result of a complex interaction between systemic host impairment and local construct‐related biological limitations, rather than as a failure mechanism attributable to a single factor. Therefore, our hypothesis, based on clinical experience and case analysis, suggests that the osteogenic capacity might be significantly reduced due to local factors (revision situation, chronic infection, heat caused by curing of the cement) as well as systemic factors (patient inherent comorbidities). Thus, incorporation of impacted bone grafts may be significantly hampered when used in conjunction with PMMA cement. The interposition of cement may impede vascular invasion and cellular migration, effectively sealing off the graft from the host bone environment and leading to a biologically inert construct.

Histological studies and micro‐CT analyses of explanted constructs show some viability for up to the first year, with only partial or superficial remodeling, with large areas of nonviable graft material remaining many years postimplantation [7, 8]. This undermines the intended goal of bone stock restoration. Without full biological incorporation, the mechanical strength of the graft‐cement construct may deteriorate over time, leading to loosening or collapse, particularly under cyclical loading [9]. This may explain the rates of late aseptic loosening observed in long‐term studies. In line with these observations, the described radiographic and histomorphological findings are closely aligned, as radiopaque PMMA cement inclusions correspond to conglomerates of cement and fragmented cancellous bone consistently devoid of osteocytes, indicating nonviable graft tissue. This spatial concordance suggests that such radiopaque regions represent biologically inert constructs with limited regenerative potential and may contribute to late loosening in the long term [10].

Another important local factor is chronic low‐grade infection. *C. acnes* is a low‐virulence, biofilm‐associated organism that may cause subtle and diagnostically challenging periprosthetic joint infections and may also be detected during revisions initially considered aseptic [11–13]. Biofilm‐associated low‐grade infections can maintain chronic inflammation at the bone‐implant or bone‐cement interface and may compromise osteogenesis, vascular invasion, and graft remodeling. Incompletely incorporated and devitalized impacted graft material may therefore serve as a protected niche for bacterial persistence. In our patient, *C. acnes* was detected in the preoperative aspiration, and histological analysis showed bone marrow edema and mineral‐salt precipitation, which were interpreted as signs of chronic irritation or infection. These findings support the possibility that infection contributed to graft nonviability. Nevertheless, causality cannot be established from a single case: Infection may have been a primary driver of loosening, a cofactor that accelerated failure of a biologically compromised construct, or a secondary colonization of an already loosened implant.

Given these limitations, alternative approaches are increasingly considered in contemporary revision THA. Uncemented modular revision systems using highly porous metal cups and augments, including porous tantalum or titanium constructs, aim to provide immediate mechanical stability while enabling osseointegration and have shown encouraging midterm to long‐term outcomes in complex acetabular bone loss [14, 15]. For severe uncontained defects, pelvic discontinuity, or distorted pelvic anatomy, cup‐cage constructs, custom triflange components, or patient‐specific 3D‐printed implants may be considered. Recent midterm series of porous titanium cup‐cage reconstructions report high implant survivorship, consistent radiographic osseointegration, and meaningful functional improvement in complex acetabular bone loss [16, 17]. In parallel, biologically active or osteoconductive graft alternatives, including biphasic injectable bone graft substitutes and potential biological adjuvants, have been explored to support defect filling and remodeling [18].

Nonetheless, the Slooff technique may still have a role in selected patients and healthcare systems, particularly where immediate mechanical stability and simplicity and/or cost‐effectiveness are priorities, and long‐term expectations are limited due to age or comorbidity.

## 4. Conclusion

This case highlights the failure of a Slooff acetabular reconstruction 15 years after implantation in a multimorbid elderly patient complicated by low‐grade periprosthetic joint infection. The case is subject to important confounders—including hemophilia A, dialysis‐dependent kidney disease, obesity, COPD, and prolonged critical illness—which may have influenced the clinical course and healing potential and should therefore be considered when interpreting the long‐term graft appearance. Histological analysis of the explant indicates the absence of viable bone and only partial remodeling, underscoring concerns about the long‐term biological integration of impacted allografts stabilized with cement. However, these findings should not be interpreted as proof of isolated technique‐related failure, but rather as evidence of a multifactorial process involving systemic host factors, local construct‐related limitations, mechanical loading, and chronic low‐grade infection. Although the Slooff technique remains a valuable and accessible option for selected cases of acetabular protrusion, its limitations—particularly the risk of incomplete incorporation, late loosening, and potential susceptibility to low‐grade infection—must be carefully weighed. Future strategies may focus on biologically active alternatives, uncemented fixation concepts, and patient‐tailored reconstructive approaches to optimize long‐term outcomes. However, the conclusions are limited by a single case design, substantiating further studies to clarify the associated risks and alternative strategies.

## Author Contributions

L. P. Weimer and G. Beckers contributed equally to this work.

## Funding

Open access funding was enabled and organized by Projekt DEAL.

## Ethics Statement

Ethics approval was not obtained due to clinical standard treatment.

## Consent

Written informed consent was obtained from the patient. A copy of the written consent is available for review by the editor of this journal. Research was performed as part of the employment of all authors.

## Conflicts of Interest

The authors declare no conflicts of interest.

## Supporting Information

Additional supporting information can be found online in the Supporting Information section.

## Supporting information


**Supporting Information 1** Case‐specific CARE checklist.

**Supporting Information 2** A structured patient timeline.

## Data Availability

The data that support the findings of this study are available on request from the corresponding author. The data are not publicly available due to privacy or ethical restrictions.
