# Supplementary material for: Histological Analysis of Acetabular Bone Impaction Grafting According to Slooff 15 Years After Implantation
Source: Case Rep Orthop. 2026 Sep 17;2026:2671931. doi: 10.1155/cro/2671931 (PMC13583925; doi:10.1155/cro/2671931)
Supplement: Supplementary file 1 [file CRO-2026-2671931-s001.pdf]

CARE Checklist of information to include when writing a case report

CARE case report guidelines

(cc) BY-NC-ND

Topic	Item	Checklist item description	Reported on Line
Title	1	The diagnosis or intervention of primary focus followed by the words "case report"	1
Key Words	2	2 to 5 key words that identify diagnoses or interventions in this case report, including "case report"	26
Abstract (no references)	3a	Introduction: What is unique about this case and what does it add to the scientific literature?	10-12
	3b	Main symptoms and/or important clinical findings	12-14
	3c	The main diagnoses, therapeutic interventions, and outcomes	13-15
	3d	Conclusion—What is the main "take-away" lesson(s) from this case?	19-25
Introduction	4	One or two paragraphs summarizing why this case is unique (**may include references**)	34 - 45
Patient Information	5a	De-identified patient specific information.	41-45
	5b	Primary concerns and symptoms of the patient.	47-53
	5c	Medical, family, and psycho-social history including relevant genetic information	47-56
	5d	Relevant past interventions with outcomes	53-89
Clinical Findings	6	Describe significant physical examination (PE) and important clinical findings.	47 - 89
Timeline	7	Historical and current information from this episode of care organized as a timeline	Supplemental material 1
Diagnostic Assessment	8a	Diagnostic testing (such as PE, laboratory testing, imaging, surveys).	47 - 89
	8b	Diagnostic challenges (such as access to testing, financial, or cultural)	n/a
	8c	Diagnosis (including other diagnoses considered)	59-63
	8d	Prognosis (such as staging in oncology) where applicable	n/a
Therapeutic Intervention	9a	Types of therapeutic intervention (such as pharmacologic, surgical, preventive, self-care)	57-89
	9b	Administration of therapeutic intervention (such as dosage, strength, duration)	57-89
	9c	Changes in therapeutic intervention (with rationale)	57-89
Follow-up and Outcomes	10a	Clinician and patient-assessed outcomes (if available)	85-87
	10b	Important follow-up diagnostic and other test results	90-149
	10c	Intervention adherence and tolerability (How was this assessed?)	n/a
	10d	Adverse and unanticipated events	n/a
Discussion	11a	A scientific discussion of the strengths AND limitations associated with this case report	152 - 226
	11b	Discussion of the relevant medical literature **with references**.	152 - 226
	11c	The scientific rationale for any conclusions (including assessment of possible causes)	152 - 225
	11d	The primary "take-away" lessons of this case report (without references) in a one paragraph conclusion	228 - 244
Patient Perspective	12	The patient should share their perspective in one to two paragraphs on the treatment(s) they received	n/a
Informed Consent	13	Did the patient give informed consent? Please provide if requested	Yes ☒ No ☐
