# Supplementary material for: Histological Analysis of Acetabular Bone Impaction Grafting According to Slooff 15 Years After Implantation
Source: Case Rep Orthop. 2026 Sep 17;2026:2671931. doi: 10.1155/cro/2671931 (PMC13583925; doi:10.1155/cro/2671931)
Supplement: Supplementary file 2 [file CRO-2026-2671931-s002.docx]

| **Month / Year** | **Event / Intervention** | **Details** |
| --- | --- | --- |
| 1996 | Primary right Total hip arthroplasty (THA) | Initial implantation of right total hip prosthesis |
| 2001 | Follow‑up radiograph | Anteroposterior pelvic radiograph after primary THA. |
| 2009 | Slooff acetabular reconstruction (right) and left cup revision | Homologous impacted bone graft with cement on right; cup revision on left for aseptic loosening. |
| 2011 | Follow‑up radiographs | Pelvic radiographs showing post‑revision status. |
| 2014 | Radiographic progression | Gradual loosening of Slooff construct and increasing cup inclination on the right. |
| December 2024 | Clinical presentation | Acute immobilizing right hip pain; suspected implant failure confirmed by X‑ray and CT. |
| December 2024 | Diagnostic workup | Joint aspiration: leukocytes 9,333/µl; Cutibacterium acnes indicating chronic PJI. |
| December 2024 | Surgical revision | Explantation of THA and conversion to Girdlestone situation; Slooff graft explanted. |
| December 2024 | Respiratory/infectious complication | Severe influenza with secondary bacterial infection; intubation and intensive care required. |
| December 2024 | GI diagnostics | Colonoscopy/gastroscopy exclude upper GI bleeding; suspicion of ischemic colitis. |
| January 2025 | Critical event | In‑hospital cardiac arrest due to hypoxia in the context of aspiration pneumonia. |
| January 2025 | Abdominal surgery | Left hemicolectomy, transverse colon resection, and enterostoma formation for ongoing bleeding. |
| January 2025 | Ascites workup and infection | Ascites puncture: elevated neutrophils; Enterococcus faecium and Candida albicans; broad anti‑infective therapy. |
| February 2025 | Clinical outcome | Death approximately 4 weeks after explantation despite maximal intensive care. |
